# Inhibitory Effects of Some Hydrocolloids on the Formation of Advanced Glycation End Products and Heterocyclic Amines in Chemical Models and Grilled Beef Patties

**DOI:** 10.3390/polym15193914

**Published:** 2023-09-28

**Authors:** Hongfei Du, Tiantian Huang, Maomao Zeng, Qingwu Shen, Ye Jiao, Wei Quan

**Affiliations:** 1 College of Food Science and Technology, Hunan Agricultural University, Changsha 410128, China; dhf2057164120@aliyun.com (H.D.); yaoyao3153@aliyun.com (Q.S.); 2 State Key Laboratory of Food Science and Technology, Jiangnan University, Wuxi 214122, China; 3 School of Food Science and Bioengineering, Changsha University of Science and Technology, Changsha 410114, China; jy_fuyao@126.com

**Keywords:** hydrocolloids, chemical model system, heterocyclic amines, advanced glycation end products, grilled beef patties

## Abstract

Effectively inhibiting the formation of heterocyclic amines (HAs) and advanced glycation end products (AGEs) is crucial to human health. In the present study, chemical model systems were used to evaluate the inhibitory effects of seven hydrocolloids on HA and AGE formation. The results showed that hydrocolloids effectively inhibited the formation of two major AGEs. However, their inhibitory action against HA formation showed unexpected results, wherein alginic acid, carrageenan and konjac glucomannan promoted the formation of 2-Amino-1-methyl-6-phenylimidazo [4,5-b]pyridine (PhIP), harmane, norharmane and 2-amino-3,8-dimethyl-imidazo [4,5-f]-quinoline (MeIQx). Only chitosan and pectin showed significant inhibitory effects on HAs, reducing HA levels by 34.5–56.3% and 30.1–56.6%, respectively. In grilled beef patties, the addition of 1.5% chitosan and pectin significantly decreased AGE and HA content by 53.8–67.0% and 46.9–68.1%, respectively. Moreover, it had a limited impact on quality and sensory properties. Further mechanism studies conducted in model systems revealed that chitosan and pectin decreased the formation of key intermediates of AGEs and HAs. These findings suggest that chitosan and pectin are powerful inhibitors against AGE and HA formation with minimal impact on food quality. Therefore, their application in meat preparation and processing could effectively decrease human dietary exposure to HAs and AGEs.

## 1. Introduction

Currently, the consumption of high-temperature processed foods with high protein and fat content is increasing [1]. In the baking process, the Maillard reaction produces desirable color and flavor compounds that enhance sensory qualities for consumers [2]. However, many studies have reported that heat treatment leads to the formation of some toxic byproducts, including advanced glycation end products (AGEs) and heterocyclic amines (HAs) [3,4]. AGEs are complex glycated compounds formed by the reaction of carbonyl groups with free amino groups [5,6]. Major AGEs including Nε-carboxymethyllysine (CML) and Nε-carboxyethyllysine (CEL) have been identified in various food products [7]. Many studies confirmed that the in vivo consumption of these compounds increases serum AGE levels in humans and is associated with inflammation, cancer, atherosclerosis and diabetes through the AGE-RAGE pathway [8,9,10]. HAs are a class of hazardous polycyclic aromatic compounds that are produced through the Maillard reaction. This reaction usually occurs when creatinine, carbohydrates and amino acids are heated at high temperatures [11,12]. The IARC has classified some HAs as Class 2A and 2B human carcinogens [13] as they can generate DNA adducts and increase the risk of cancers, especially liver, colon and prostate cancer [14].

Thus, strategies to inhibit AGE and HA formation in food products need to be developed to attenuate the health risks associated with dietary exposure. Recently, researchers have sought to use food additives as inhibitors to reduce the AGE and HA content in food products. Extensive research has been conducted on extracts derived from plants or phytochemicals, investigating their remarkable inhibitory effects and the potential advantages they offer for health. Furthermore, these compounds have gained recognition as a highly propitious method for diminishing the health hazards related to HAs and AGEs [15,16]. In previous studies, pepper, ginger and kaempferol were found to inhibit AGE and HA formation in a chemical model and grilled beef patties [17,18,19]. However, the use of phenolic compounds in foods remains severely limited due to their potential negative impact on the texture and sensory attributes of the product. Additionally, their poor solubility in food materials further hampers their application [20,21].

Hydrocolloids are widely used in the food industry with various functional properties [22,23]. Currently, some hydrocolloids have attracted considerable attention because of their ability to reduce the formation of thermally induced toxic compounds [24]. According to Zhang and colleagues (2020), the formation of PhIP in chemical model systems can be effectively suppressed by alginic acid, CMC-Na and chitosan. Among these, CMC-Na exhibited the highest inhibitory activity against HAs in roasted beef patties [25]. Similarly, Oz and associates observed that chitosan was successful in curbing the formation of HAs in steak and cooked meatballs [26]. Moreover, alginic acid, pectin, CMC-Na and chitosan could dose-dependently inhibit CML and CEL formation in chemical models and fish patties [27]. Meanwhile, other studies showed that chitosan and κ-carrageenan in cakes exhibited the highest inhibitory activity on the formation of fluorescent and nonfluorescent AGEs [28,29].

The formation mechanisms and inhibition methods of HAs and AGEs, generated through heating food, have been investigated separately but not synchronously [12,30]. Few studies have simultaneously explored the generation profile and inhibition methods of these two compounds, leaving a gap in the research that fails to meet the demand for public food safety [18]. To the best of our knowledge, no studies have used hydrocolloids as HA and AGE inhibitors. Therefore, this study investigated the effects of different hydrocolloids (e.g., alginic acid, chitosan, carrageenan, KGM, pectin, gellan and xanthan gum) on HA and AGE formation in chemical models and grilled beef patties. Additionally, the effects of specific hydrocolloids on intermediate compounds of AGE and HA were further assessed using chemical models to elucidate their inhibitory mechanisms. Finally, the changes in the proximate composition, texture profile and sensory quality of grilled beef patties were evaluated. The results of this study provide valuable information for the development of new combinations to enhance food safety and quality, as well as for identifying promising inhibitors of HA and AGEs.

## 2. Materials and Methods

### 2.1. Materials and Chemicals

Beef was obtained from a local market in Changsha, China. Hydrocolloids including alginic acid, chitosan, carrageenan, xanthan gum, konjac glucomannan (KGM), pectin and gellan gum were purchased from Aladdin (Shanghai, China). Chemical standards of HAs: PhIP, IQ, MeIQ, MeIQx, 4,8-DiMeIQx, IQx, Harmane and Norharmane were supplied by Santa Cruz Biotechnology, Inc. (Santa Cruz, CA, USA). Chemical standards of AGEs, *N^ε^*-(carboxymethyl)lysine (CML), d_4_-CML, *N*^ε^-(carboxyethyl)lysine (CEL) and d_4_-CEL, were obtained by Santa Cruz Biotechnology Co. (Paso Robles, CA, USA). The standards of lysine, phenylalanine, creatinine, glucose, threonine, tryptophan, phenylacetaldehyde, 2,5-dimethylpyrazine, glyoxal, methylglyoxal, O-phenylendiamine, 2,3-hexanedione and nonafluoropentanoic acid (NFPA) were purchased from J&K Scientific Co, Ltd. (Beijing, China). Mass spectral grade methanol and acetonitrile were purchased from Merck KGaA (Darmstadt, Germany). Oasis MCX (3 cc/60 mg) cartridges were purchased from Waters (Milford, MA, USA). The chemicals were all analytical grade and purchased from Sinopharm Chemical Reagent Co., Ltd. (Shanghai, China).

### 2.2. Inhibitory Effects of Hydrocolloids on HA and AGE Formation in Chemical Model Systems

HA and AGE chemical model systems were established in accordance with previously reported methods [31,32,33] with some modifications to simulate the formation of IQ-type HAs, quinoxaline-type HAs and AGEs in grilled beef patties. The experimental groups consisted of alginic acid, chitosan, carrageenan, xanthan gum, KGM, pectin and gellan, which were added to the mixtures to achieve final concentrations of 0.1%, 0.5% and 1.5% (*w*/*w*). These hydrocolloids were dissolved in 10 mL of a 0.2 mol/L phosphate buffer solution, along with glucose (0.2 mmol), creatine (0.4 mmol), phenylalanine (0.4 mmol), lysine (0.4 mmol), tryptophan (0.2 mmol) and threonine (0.4 mmol). After heating at 175 °C for 20 min, the reaction mixture was cooled immediately to terminate the reaction.

### 2.3. High-Performance Liquid Chromatography with Tandem Mass Spectrometry (HPLC-MS/MS) Analysis of AGEs from Chemical Model Systems

Based on our previous study [34], 150 μL reacted solution was mixed with 150 μL of internal standard solution (500 ng/mL d_4_-CML and d_4_-CEL). Then, 5 μL mixed solution was separated using HPLC-MS/MS. Analysis of CML and CEL was performed on a Waters 2695 system equipped with an X-Bridge C18 column (2.1 × 100 mm, 3.5 μm, Milford, MA, USA). A flow rate of 0.3 mL/min was used under gradient elution with acetonitrile and NFPA (5 mM) as the mobile phase. The gradient elution procedure and MS conditions were the same as in our previous studies [34]. With reference to an external standard calibration curve, isotopically labeled internal standards were used to quantify CML and CEL.

### 2.4. Ultra-Performance Liquid Chromatography with Tandem Mass Spectrometry (UPLC-MS/MS) Analysis of HAs from Chemical Model Systems

A total volume of 5 mL of the reaction mixture underwent extraction using 5 mL of ethyl acetate via vortexing for a duration of 5 min and ultrasonication for 10 min. This extraction process was iterated three times. Afterward, the combined ethyl acetate extraction solutions were concentrated to roughly 20 mL using nitrogen blowing. Subsequently, these solutions were introduced into a Waters Oasis MCX cartridge (3 mL, 60 mg) for elution and purification following our previously established procedure [18,31].

HAs were separated, identified and quantified using a Waters Acquity UPLC system (Waters, Milford, MA, USA) equipped with a triple quadrupole mass spectrometer and Acquity UPLC BEH C18 column (50 × 2.1 mm i.d., 1.7 m) at 35 °C. The gradient elution procedure and MS parameters were the same as in our previous studies [18,31].

### 2.5. Effects of Hydrocolloids on HA and AGE Formation in Grilled Beef Patties

Based on our previous study, slight modifications were made to analyze the impact of hydrocolloids on the formation of HA and AGE in grilled beef patties [18,19]. After thoroughly mixing with a hydrocolloid (1.5% *w*/*w*), beef patties (40 ± 0.1 g) were prepared using a petri dish (Φ 6 cm × 1.5 cm) to ensure uniformity. Then, the patties were roasted using a RATIONAL SCC 61 E Self Cooking Center oven (Landsberg, Munich, Germany) at 225 °C for 10 min on each side.

### 2.6. Determination of the Inhibitory Effect of Hydrocolloids on HA and AGE Formation in Grilled Beef Patties

Extraction of HAs from grilled beef patties: According to our previously described method [17,19], beef powder was mixed with 2M NaOH solution and extracted using 20 mL of ethyl acetate. After being centrifuged at 3000× *g* for 10 min, the supernatant was collected and concentrated to approximately 5 mL for solid-phase extraction, which was conducted according to our previously described protocol.

Extraction of AGEs from grilled beef patties [17,18]: 30 mg of beef patties was defatted first using n-hexane. Then, the dry defatted powder was treated with 1.5 mL of 0.2 M borate buffer and 1.5 mL of 1 M sodium borohydride for 10 h at 4 °C and was hydrolyzed with 6 mL HCl at 110 °C for 12 h. The filtered hydrolysate underwent dilution to achieve a 10 mL final volume with ultrapure water. To act as internal standards, 100 μL of d_4_-CML and d_4_-CEL (at a concentration of 500 ng/mL) were added. Subsequently, the mixture was dried utilizing a rotary evaporator at 50 °C under a vacuum. After drying, the protein hydrolysate was reconstituted with 2 mL of water. Prior to CML and CEL analysis, the reconstituted hydrolysate was subjected to purification using an MCX cartridge (3 mL, 60 mg), following the previously described protocol.

Finally, HPLC-MS/MS of AGEs and UPLC-MS/MS analysis of HAs were performed as described in Section 2.3 and Section 2.4, respectively.

### 2.7. Quality Analysis (Composition, Color, Texture and Cooking Loss) and Sensory Evaluation of Grilled Beef Patties Added with Hydrocolloids

(a) The proximate composition (protein, fat, ash and moisture content) of raw and cooked samples was determined according to AOAC procedures. The moisture content was determined by drying (105 °C) in a MemmertUF30 universal oven (Schwabach, Germany); the total ash by incineration (at 550 °C) using a laboratory Heraeus M110 muffle furnace (Heraeus, Hanau, Germany); the total protein content (N × 6.25) using the Kjeldahl method; and the fat content using the Soxhlet method (with n-hexane as a solvent) using the Büchi Extraction System B-811 (Flawil, Switzerland).
(b) The meat pH was measured using a digital pH meter equipped with a penetration glass electrode (Elmetron, Zabrze, Poland) directly in the grilled meat patties.
(c) The cooking loss (CL) of the grilled beef patties was expressed as the percentage difference between the initial weight of the raw meat sample and the cooked sample in the electric grill.
(d) The texture profile of the grilled beef patties was analyzed at room temperature (20–24 °C) using a TA-XT plus texture analyzer (Godalming, UK) equipped with a P/50 cylinder probe (50 mm). The roasted patties were cut into small cubes (1 × 1 × 1 cm) and the hardness, springiness, gumminess, cohesiveness and chewiness were measured using the following parameters: 3 mm/s for the pre-test, 2 mm/s for the test and 3 mm/s for the post-test. The trigger force was set at 5.0 g with a 50% strain for 5 s [19].
(e) The color of the grilled beef patties was measured using a CR-400 colorimeter (Konica Minolta, Japan) portable spectrophotometer with a pulsed xenon lamp and 8 mm aperture size. The results (illuminant D65, 10° Standard Observer) were given in the CIE L*a*b* color space including the following spectral values: L*, (lightness), a* (redness) and b* (yellowness). At least three readings at different points of the sample were taken each time and the mean value was calculated for each replication.
(f) The sensory attributes of the grilled beef patties were evaluated by ten panelists of both sexes (nonsmokers, between 25 and 40 years of age) who were experienced in meat sensory analysis and instructed using a nine-point structured hedonic scale [35]. Five different sensory properties, namely appearance, flavor, tenderness, texture and overall acceptability, were assessed.

### 2.8. Determination of Dicarbonyl Compounds in Chemical Model Systems

Initially, a reaction was conducted at room temperature for 12 h in the dark between 200 μL of o-phenylenediamine (5 mM) and an equal volume of the sample. To ensure accuracy, an internal standard of 200 μL of 1 μg/mL 2,3-hexanedione was included. Following derivatization, the sample was diluted with distilled water to achieve a final volume of 2 mL. Next, the prepared samples were loaded onto an HLB column (3 mL, 60 mg) and subjected to a thorough cleaning process before HPLC-MS/MS analysis. This experimental procedure adheres to our previously published protocol [32,34].

HPLC-MS/MS analysis for GO and MGO was also performed according to our previously described protocol [32,34], in which separation was conducted on a Shimadzu C18 column (2.1 mm × 100 mm, 3 μm), and the mobile phase comprised methanol (solvent A) and 0.1% formic acid in water (solvent B). The gradient program, MS conditions and MRM mode were the same as in our previous studies [32,34].

### 2.9. Determination of Phenylacetaldehyde and 2,5-Dimethylpyrazine in Chemical Model Systems

The determination of phenylacetaldehyde and 2,5-dimethylpyrazine levels, which are two other important intermediate compounds of HAs, primarily refers to [31]. In detail, phenylacetaldehyde extraction involved the mixing of 5 mL of water and 20 mL of ethyl acetate with the sample solutions in separate funnels. Following separation, the ethyl acetate layers underwent filtration before injection into the gas chromatography (GC) system. GC detection was conducted using an Agilent 7820A GC system (Agilent Technologies, Inc., Santa Clara, CA, USA). The headspace autosampler and GC parameters were the same as in our previous study.

Moreover, solid-phase microextraction coupled with gas chromatography-mass spectrometry were used to test 2,5-dimethylpyrazine levels, the injector temperature and the detector temperature, and the flow rates of the fuel gas and oxidant gas were the same as those used in the detection of phenylacetaldehyde.

### 2.10. Statistical Analysis

Three independent repeated experiments were carried out and the experimental results were expressed as the average standard deviation. In addition, the relative amount of HA, AGE or the intermediate compound levels in the grilled beef patties or chemical model systems were calculated using that of the control. Data were analyzed using analysis of variance to find significant differences between treatments (*p* < 0.05 means statistical significance) using Statistix 9.0 software.

## 3. Results and Discussion

### 3.1. Inhibitory Effects of Hydrocolloids on HA Formation in Chemical Model Systems

**Figure 1:**
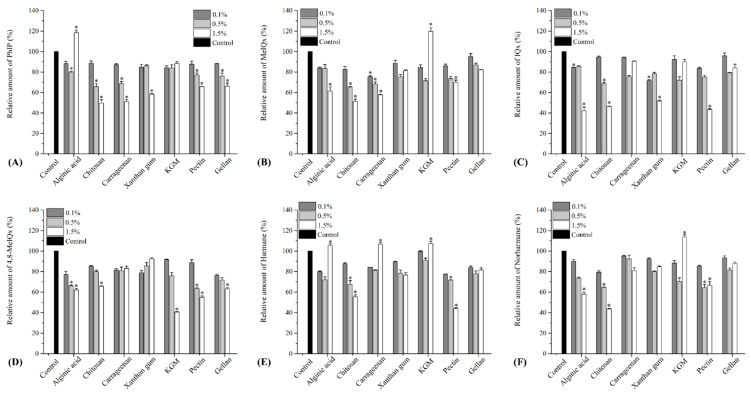
Relative amount of main heterocyclic amies (**A**) PhIP, (**B**) MeIQx, (**C**) MeIQ, (**D**) 4,8-DiMeIQx, (**E**) Harmane and (**F**) Norharmane in model systems treated with different hydrocolloids. Values are expressed as mean ± SD (*n* = 3); * indicates significant differences (*p* < 0.05).

According to previous studies, PhIP and MeIQx are the most abundant HAs in cooked food. An IQ-type and quinoxaline-type HA model system was chosen to evaluate the inhibitory effects of seven hydrocolloids against HA formation at various addition levels (0.1%, 0.5% and 1.5%). Six kinds of HAs, including PhIP, MeIQx, MeIQ, 4,8-MeIQx, harmane and norharmane, were detected. As shown in Figure 1, seven hydrocolloids inhibited HA formation by approximately 4.1–28.3% at a 0.1% addition level, with xanthan gum significantly inhibiting MeIQx and 4,8-MeIQx formation (28.3% and 21.2%, respectively, relative to the control). Chitosan, pectin and gellan significantly inhibited norharmane, 4,8-MeIQx and harmane formation (20.7%, 22.4% and 23.8%, respectively, relative to the control). In addition, KGM exhibited a limited inhibitory effect on HAs, and the content of several HAs was not significantly reduced compared to that of the control group.

At a 0.5% addition level, the inhibitory effect of chitosan and pectin against HA formation was significantly increased, whereas that of alginic acid, KGM and carrageenan did not increase. The inhibitory effect of chitosan and pectin on HA formation increased from 5.4–20.7% and 11.5–23.4% to 20.1–35.3% and 23–36.5%, respectively. When the addition level was increased to 1.5%, the inhibitory effect of hydrocolloids on HA formation showed significant changes. Although alginic acid significantly reduced the formation of four types of HAs by 37.9–57.7%, it unexpectedly increased the formation of PhIP and harmane by 5.4% and 18.3%, respectively. Similarly, carrageenan did not exhibit a significantly increased inhibitory effect on HAs, except for PhIP and MeIQx. Moreover, it even promoted the formation of harmane (6.6% relative to the control). KGM exhibited the worst inhibitory effect on HAs, except for 4,8-MeIQx. Not only did it not significantly reduce the content of other HAs, but it even significantly promoted the formation of MeIQx, harmane and norharmane (19.6%, 7.1% and 13.6%, respectively, relative to the control). Although gellan and xanthan gum did not promote the formation of HAs, their inhibitory effects on HAs did not exhibit a dose-dependent trend. Only chitosan and pectin showed significant inhibitory effects on all six HAs, reducing HA levels by 34.5–56.3% and 30.1–56.6%, respectively. The addition of levels of hydrocolloids exhibited varied inhibitory effects but only chitosan and pectin decreased the formation of all HAs in a dose-dependent manner. Furthermore, the dose–response relationship data suggested that chitosan and pectin are promising inhibitors of HA formation at relatively higher addition levels.

As frequently used stabilizers in food processing, the inhibitory effects of hydrocolloids against HA formation have attracted considerable attention. In line with our findings, Yang et al. found that the addition of 1% carboxymethylcellulose, κ-carrageenan, alginic acid and pectin in PhIP chemical models could effectively decrease the level of PhIP by 47–54% [36]. Zhang et al. evaluated the effects of six hydrocolloids on the formation of major HAs and they observed that CMC-Na and chitosan exhibited the strongest inhibitory effect on HA formation at higher addition levels [25]. Other studies have also reported that CMC-Na, chitosan and xanthan gum could inhibit PhIP formation [37]. Similar to previous studies, the inhibitory effects of hydrocolloids on HA formation can be explained by the following reasons: carboxyl groups of hydrocolloids can interfere with the decarboxylation process, which may decrease the formation of HA [38]. Furthermore, the presence of hydrocolloids rich in amino acids can cause a reaction with reactive carbonyl compounds, which play a role in the formation of HAs, resulting in a substantial decrease in the levels of HA. Additionally, the inhibitory effects on the formation of HAs can vary due to the different functional groups and structures of hydrocolloids, which possess nucleophilic properties [25,36].

### 3.2. Inhibitory Effects of Hydrocolloids on AGE Formation in Chemical Model Systems

**Figure 2:**
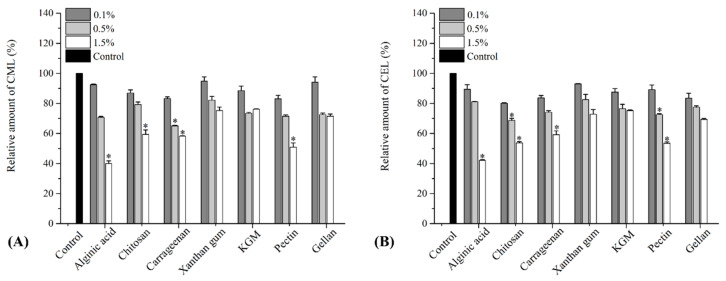
Relative amount of advanced glycation end products (**A**) CML, (**B**) CEL in model systems treated with different hydrocolloids. Values are expressed as mean ± SD (*n* = 3); * indicates significant differences (*p* < 0.05).

CML and CEL are typical AGEs widely found in meat, fish and dairy products [7,39]. As shown in Figure 2, all seven hydrocolloids significantly reduced CML and CEL formation at each addition level (*p* < 0.05), which is similar to the inhibitory effects of polyphenols, such as resveratrol on CML and CEL reported by other researchers [39]. For example, at a 0.1% addition level, alginic acid and xanthan gum decreased CML content by 7.6% and 5.2%, respectively, and CEL content by 10.6% and 7.0%, respectively (Figure 2A). Moreover, chitosan, carrageenan and pectin exhibited a stronger inhibitory effect against CML and CEL formation than alginic acid and xanthan gum, decreasing CML content by 13.1%, 16.8% and 16.9%, respectively, and CEL content by 19.9%, 16.3% and 10.9%, respectively. At a 0.5% addition level, the hydrocolloids further inhibited CML and CEL formation in the model systems by 17.5–34.9% and 17.8–31.3%, while carrageenan and chitosan exhibited the highest inhibitory effect against CML and CEL formation, respectively. With the further increase in the additive level, alginic acid, chitosan, carrageenan and pectin inhibited the generation of both CML and CEL in a dose-dependent manner in the chemical system, which significantly decreased the content of CML and CEL by 40.6–60.0% and 40.8–57.9%, respectively.

These findings align with previous reports indicating that multiple hydrocolloids have the ability to inhibit AGE formation in chemical model systems. For example, Xu et al. confirmed that alginic acid, pectin, CMC-Na and chitosan could significantly inhibit CML and CEL formation in chemical models, with pectin and alginic acid being the most effective inhibitors [27]. In another study, chitosan effectively inhibited AGE formation in a BSA–fructose model [28]. Moreover, carrageenan has been reported to exhibit the highest inhibitory activity on AGE formation in the lysine–glucose model and cakes [29]. These findings demonstrated that hydrocolloids are potential AGE inhibitors. Some studies reported that the products derived from the Maillard reaction of lysine and polysaccharides exhibit strong antioxidant activity, which might effectively inhibit AGE formation [40]. Additionally, researchers have demonstrated that the potential inhibition mechanism of hydrocolloids on the formation of AGEs is the same as that of phenolic compounds. The nucleophilic groups (such as amino, carboxyl, sulfate and hydroxyl groups) of those hydrocolloids can directly react with intermediate compounds through electrophilic substitution, which effectively inhibits the formation of AGEs [27,41]. Meanwhile, due to the distinct nucleophilic characteristics displayed by various functional groups and structures, the inhibitory efficiency of hydrocolloids may vary depending on the specific compound. This could explain why pectin and alginic acid exhibit significantly stronger inhibitory effects against CML and CEL formation compared to KGM, gellan and xanthan gum.

### 3.3. Effects of Hydrocolloids (Chitosan or Pectin) on HA and AGE Formation in Grilled Beef Patties

Although all selected hydrocolloids could effectively reduce AGE formation, their inhibitory effects were further evaluated in grilled beef patties, a type of red meat that is the most widely consumed and easily forms a large amount of HAs and AGEs during processing. Considering that HA was only decreased by chitosan and pectin addition, in this experiment, the most effective addition level (1.5%) was selected. As shown in Table 1, chitosan and pectin significantly (*p* < 0.05) reduced the formation of CML, CEL and all six HAs in grilled beef patties compared to the control group. The addition of 1.5% chitosan and pectin significantly decreased the AGE and HA content by 53.8–67.0% and 46.9–68.1%, respectively.

Moreover, our results indicate that the inhibitory activity of chitosan and pectin against the formation of HAs and AGEs in grilled beef patties was significantly weaker compared to that observed in chemical models. For example, in grilled beef patties, chitosan and pectin reduced CML formation by 64.5% and 55.0% and CEL formation by 61.0% and 61.6%, respectively. However, in chemical models, chitosan and pectin reduced CML formation by 59.4% and 50.9% and CEL formation by 53.8% and 53.5%, respectively. Our findings were consistent with Xu et al.’s results, which suggest that the movement and interaction between molecules in grilled beef patties are more limited compared to chemical models [27]. The interaction among amino acids and sugars could potentially be restricted by the thickening and gelling properties of hydrocolloids, and further hinder the reaction between the precursors and the intermediates of AGEs and HAs. The different inhibitory activities of the hydrocolloids against the formation of AGEs and HAs in both grilled beef patties and chemical models are a result of the abovementioned variations [27].

### 3.4. Influence of Hydrocolloids (Chitosan or Pectin) on the Selected Quality and Sensory Properties of Grilled Beef Patties

As chitosan and pectin showed strong inhibitory activities against HA and AGE formation in grilled beef patties, we further assessed their effects on the quality and sensory properties of grilled beef patties. As shown in Table 2, the pH values; cooking loss; and protein, ash and fat content of beef patties from the control group were 5.52, 51.3%, 44.3 g/100 g, 4.36% and 3.42%, respectively, which were comparable to the values reported in previous studies. Compared to the control group, the pH value and protein, fat and ash content in the beef patties treated with hydrocolloids were changed nonsignificantly. However, the cooking loss values showed significant changes (*p* < 0.05) between the control group and the patties treated with hydrocolloids, which decreased from 51.3% to 48.5% and 47.2%. The TPA results of grilled beef patties are presented in Table 3. As can be seen, chitosan and pectin do not change the springiness, cohesiveness and gumminess of grilled beef patties, which showed similar results to previous studies [27]. However, the hardness decreased with the addition of pectin but not chitosan. Moreover, the addition of chitosan and pectin slightly decreased the chewiness from 25.8 to 21.8 and 21.3, respectively. Although studies have reported that hydrocolloids have water-binding abilities, stabilizing emulsion and modifying the gelation process, and further forms a porous network held between protein–gel matrix and the fat globules in the meat product system, which then plays a key role in the textural parameters of meat products. But our results indicated that hydrocolloids had a limited impact on the textural properties of grilled beef patties, which might relate to the low addition level of hydrocolloids and the interaction between hydrocolloids and active carbonyl compounds. In addition, the effects of different hydrocolloids on the color of grilled beef patties are shown in Table 4. No significant differences in L*, a* and b* values were observed between the chitosan, pectin and control groups, indicating that these two hydrocolloids did not change the brightness of the grilled beef patties.

**Figure 3:**
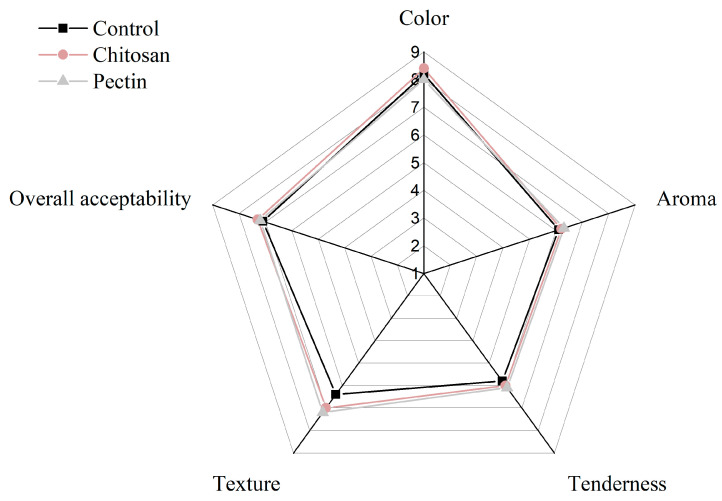
Impact of 1.5% chitosan and pectin on sensory quality of grilled beef patties. 1–9 means the score of sensory quality analysis.

Additionally, the sensory characteristics of the grilled beef patties were also evaluated and the results are shown in Figure 3. Although the sample added with chitosan or pectin had a higher texture score, there were no significant differences between the control group and the hydrocolloid group in terms of overall acceptability. Therefore, these two hydrocolloids effectively reduced AGE and HA formation without altering the quality and sensory characteristics of the grilled beef patties.

### 3.5. Effects of Hydrocolloids (Chitosan or Pectin) on HA and AGE Intermediates in Chemical Models

**Figure 4:**
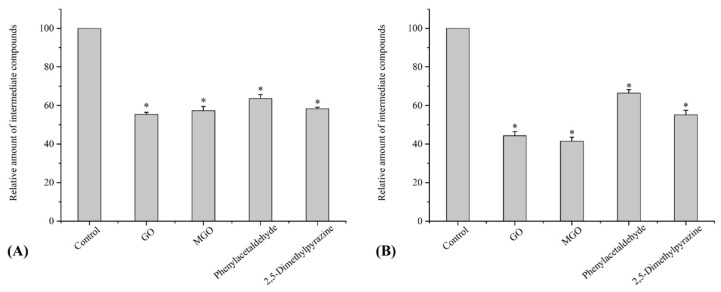
Relative amount of intermediate compounds in model systems treated with (**A**) 1.5% chitosan and (**B**) pectin. Values are expressed as mean ± SD (*n* = 3); * indicates significant differences (*p* < 0.05).

As chitosan and pectin showed the most potent inhibitory activity against HA and AGE formation in chemical models and grilled beef patties, the effects of 1.5% chitosan or pectin on the content of intermediates in chemical model systems were examined to further investigate the inhibition mechanism of HA and AGE formation. Based on previous studies [12,16,32], some key intermediates of HAs and AGEs, including GO, MGO, phenylacetaldehyde and 2,5-dimethylpyrazine, were measured. As illustrated in Figure 4, chitosan and pectin displayed inhibitory activity against the formation of all intermediates compared to the control sample (*p* < 0.05), with pectin showing stronger inhibitory activity on GO and MGO than chitosan (55.7% and 58.5%, respectively). Our results were consistent with those of previous studies, which found that hydrocolloids could inhibit PhIP formation through the consumption effect of precursors of reactive dicarbonyl compounds [36]. In addition, hydrocolloids could conjugate with GO and MGO or react with phenylacetaldehyde and then block AGE and HA formation following the reaction of dicarbonyl compounds and phenylacetaldehyde.

## 4. Conclusions

The inhibitory effects of seven hydrocolloids on HA and AGE formation were investigated in chemical model systems and grilled beef patties. Our findings indicate that the addition of chitosan or pectin effectively reduced HA and AGE formation in chemical models and grilled beef patties. The addition of 1.5% (*w*/*w*) chitosan or pectin reduced HA and AGE formation by approximately 50% in chemical models and grilled beef patties without significantly affecting the quality and sensory properties of grilled beef patties. Further mechanism investigation pointed out that pectin and chitosan could inhibit the formation of intermediate compounds of AGEs and HAs including GO, MGO, phenylacetaldehyde and 2,5-dimethylpyrazine. The findings of the current study could assist in the development of hydrocolloid-based food additives to minimize the formation of HAs and AGEs, thereby enhancing food safety. Further mechanistic investigations of grilled beef patties are needed to confirm the inhibitory mechanisms of hydrocolloids against HAs and AGEs.

## Figures and Tables

**Table 1:** Relative amount of main heterocyclic amies and advanced glycation end products in grilled beef patties treated with 1.5% chitosan or pectin.

	PhIP (%)	MeIQx (%)	IQx (%)	4,8-MeIQx (%)
Chitosan	54.9 ± 1.36	59.3 ± 1.59	53.8 ± 0.65	67.4 ± 1.30
Pectin	68.1 ± 1.02 *	64.5 ± 1.21 *	51.5 ± 0.84 *	58.6 ± 1.56
	Harmane (%)	Norharmane (%)	CML (%)	CEL (%)
Chitosan	57.2 ± 0.61	54.3 ± 0.79 *	64.5 ± 1.84	61.0 ± 1.76
Pectin	46.9 ± 1.69 *	63.0 ± 1.88	55.0 ± 1.85 *	61.6 ± 1.25 *

* indicated significant differences (*p* < 0.05).

**Table 2:** Chemical composition, cooking loss and pH value of the grilled beef patties treated with 1.5% chitosan or pectin.

Group	pH	Protein (g/100g)	Cooking Loss (%)	Ash (%)	Fat (%)
Control	5.52 ± 0.16	44.3 ± 1.74	51.3 ± 0.63	4.36 ± 0.07	3.42 ± 0.85
Chitosan	5.57 ± 0.21	46.7 ± 0.57	48.5 ± 0.81 *	4.52 ± 0.11	3.06 ± 0.68
Pectin	5.79 ± 0.29	47.3 ± 2.56	47.2 ± 1.15 *	4.28 ± 0.19	3.73 ± 0.64

* indicated significant differences (*p* < 0.05).

**Table 3:** Texture characteristics of the grilled beef patties treated with 1.5% chitosan or pectin.

Group	Hardness (N)	Springiness (mm)	Gumminess (N)	Cohesiveness (N)	Chewiness (N)
Control	90.1 ± 2.47	0.56 ± 0.03	37.1 ± 0.72	0.60 ± 0.05	25.8 ± 0.69
Chitosan	86.7 ± 3.38	0.57 ± 0.04	36.8 ± 0.59	0.65 ± 0.02	21.8 ± 0.56 *
Pectin	87.4 ± 1.79	0.49 ± 0.09	37.9 ± 0.80	0.62 ± 0.09	21.3 ± 0.38 *

* indicated significant differences (*p* < 0.05).

**Table 4:** Color difference analysis of grilled beef patties treated with 1.5% chitosan or pectin.

Group	L	a	b
Control	40.9 ± 1.31	9.10 ± 0.41	9.10 ± 0.41
Chitosan	41.5 ± 1.64	10.3 ± 0.76	9.87 ± 0.39
Pectin	41.9 ± 1.89	9.60 ± 0.84	8.95 ± 0.56

## Data Availability

The data presented in this study are available on request from the corresponding author.
